# Reinforcement of Polylactic Acid for Fused Deposition Modeling Process with Nano Particles Treated Bamboo Powder

**DOI:** 10.3390/polym11071146

**Published:** 2019-07-04

**Authors:** Cuicui Wang, Lee Miller Smith, Wenfu Zhang, Mingpeng Li, Ge Wang, Sheldon Q. Shi, Haitao Cheng, Shuangbao Zhang

**Affiliations:** 1 Beijing Key Laboratory of Wood Science and Engineering, Beijing Forestry University, Beijing 100083, China; 2 International Centre for Bamboo and Rattan, Beijing 100102, China; 3 Department of Mechanical and Energy Engineering, University of North Texas, Denton, TX 76207-7102, USA; 4 Zhejiang Forestry Research Institute, Hangzhou 310023, China

**Keywords:** cellulose nanofibers (CNF), micro-crystalline cellulose (MCC), nano calcium carbonate (CaCO_3_), impregnation modification (IM), bamboo powder (BP), fused deposition modeling (FDM)

## Abstract

The focus of this report was to understand the tensile properties and dynamic mechanical properties of bamboo powder (BP) reinforced polylactic acid (PLA) composite filaments which were treated with nano calcium carbonate (CaCO_3_), cellulose nanofibers (CNF), and micro-crystalline cellulose (MCC) using impregnation modification technology. The storage modulus (*E*’) of nano CaCO_3_-BP/PLA, MCC-BP/PLA, and CNF-BP/PLA composite filaments increased compared with BP/PLA composite filaments before the glass transition temperature *T*_g_. When the temperature was above *T*_g_, the reinforcement effect of nano CaCO_3_, MCC, and CNF gradually became less apparent. The loss modulus (*E*’’) and loss factor (tan *δ*_max_) of the nano CaCO_3_-BP/PLA, MCC-BP/PLA, and CNF-BP/PLA composite filaments was higher than that of BP/PLA composite filaments produced by the “one-step” method. The tensile strength (TS) results showed a similar trend. Compared with the control samples, the TS of nano CaCO_3_-BP/PLA, MCC-BP/PLA, and CNF-BP/PLA composite filaments produced by the “one-step” method (and the “two-step” method) increased by 40.33% (and 10.10%), 32.35% (and −8.61%), and 12.32% (and −12.85%), respectively. The TS of nano CaCO_3_-BP/PLA, MCC-BP/PLA, and CNF-BP/PLA composite filaments produced by the “one-step” method was slightly higher than those produced by the “two-step” method. The elongation at break (EAB) of BP/PLA composite filaments was higher than that of BP/PLA samples treated with nano CaCO_3_, MCC, or CNF. The PLA and modified BP were readily accessible through a simple mixing process. The rheological investigation of such mixtures showed that nano CaCO_3_, CNF, and MCC have different effects on the processability and rheological properties of composites.

## 1. Introduction

Natural fibers are renewable materials that are derived abundant sustainable resources (e.g., bamboo, cotton, flax, hemp, jute, kenaf, sisal, ramie, pineapple, coir, etc.), which can be isolated, treated, and functionalized for a multitude of applications, for instance, polymeric matrix composites [1], etc. In polymeric matrix composites, natural fibers as a reinforcing phase, provide positive environmental benefits with respect to ultimate disposability and raw material use [2]. And natural fiber has many advantages such as low density, low cost, low energy consumption, high specific strength, and modulus, as well as a relatively reactive surface [3]. Natural fiber reinforced polymer composites have seen wide use in many industries including automotive, aerospace, electronics, construction, and interior decoration [4,5]. However, natural fiber reinforced polymer composites are used only to a limited extent in industrial practice, because the inherent polar and hydrophilic nature of natural fiber and the nonpolar performance of thermoplastics polymer, which leads to difficulties in compounding the reinforcement and the matrix. Thus, the acceptable dispersion levels and the effective composite materials cannot be obtained.

Recent research has reported that the inorganic nanoparticle impregnation is an effective method to improve the compatibility between natural fibers and polymer matrices [6]. The mechanical properties of single bamboo fibers and its composites treated with nano CaCO_3_ impregnation modification saw increased properties compared to the untreated samples [7,8], as well as that of kenaf fibers and their composites were increased by impregnation with CaCO_3_ particles [9], which has a high surface hardness (1.5–4.7 GPa) and modulus of elasticity (73.5–85.5 GPa) [10]. Currently, cellulose nanofibers are attracting particular interest in both academia and industry because of the abundant renewable nature of cellulose, and the outstanding mechanical properties of cellulose nanocrystals [3,11]. Nano-cellulose can be used for the reinforcement of polymer composites where they are able to enhance their thermal stability and mechanical properties due to nano-cellulose having a large surface area, high aspect ratio, high crystalline structure, high on-axis stiffness (Young’s modulus of 100–143 GPa) and a unique tensile strength (0.8–10 GPa) [3,12,13]. It also has been reported that nano-cellulose has a remarkable reinforcing effect on the tensile properties of bio-polymer composites [14,15,16]. Another cellulose-derived product, micro-crystalline cellulose (MCC) is a white, tasteless, and odorless crystalline powder, which can be used as a suspension- stabilizer, thickener, a filler, or binder in tablets, and a flow characteristics modifier in various formulations [17,18]. In comparison to nano CaCO_3_, the natural nanofillers has several advantages, including biosustainability, biorenewability, low production cost, and possibly lower cytotoxic and (pro-) inflammatory effects when inhaled [19]. Interestingly, cellulose nanofibers (CNF) and MCC have not been explored as polymer reinforcement that uses the impregnation modification technology to treat reinforcement.

The focus of this report is on the nano-particles treated bamboo powder/polylactic acid composite filaments for 3D printing, a technology in which a part can be built layer by layer to the desired geometry from virtual models based on computer-aided design software, digital scanning systems, or medical imaging systems. Moreover, complex parts can be built easily in reasonable time frames [20,21,22,23,24]. Presently, a large number of additive processes including fused deposition modeling (FDM), stereolithography (SLA), selective laser sintering (SLS), selective laser melting (SLM), digital light processing (DLP), laminated object manufacturing (LOM), and so on, are available for 3D printing. Tekinalp et al. [23] investigated short fiber reinforced acrylonitrile-butadiene-styrene (ABS) composites as a feedstock for 3D-printing in terms of their processibility, microstructure, and mechanical performance. Gurr et al. [25] reported an optically transparent stereolithography resin (SLR) filled with up to 30% w/w silica nanoparticles and observed an increase of the Young’s modulus of 25% at a nanofiller content of 17% w/w without significantly impacting the optical properties; a higher nanofiller increased the stiffness by another 7% but the transparency was significantly reduced.

Currently, various nano-reinforcements have been used in 3D printed materials, including nano-cellulose [26], SiO_2_ [25,27], and layered silicate [28]. This is because nano-particles have a porous structure and the pores mainly pertain to mesopores and micropores, which indicated that they have an improved specific surface [29]. However, most of them were stereolithography (SLA) 3D printed materials. Fused deposition modeling (FDM) 3D printed nano-composites have not been fully studied. In this study, PLA was used as the matrix because of high elastic modulus, relatively low *T*_g_, and the possibility in 3D-printing, moreover, using dispersed high modulus inorganic particles to fill the PLA matrix may act as an additional fixed phase [30]. The study aimed to manufacture nano-reinforced bamboo powder (BP)/polylactic acid (PLA) composite filaments with high performance successfully used in FDM process, which can provide a new approach to explore the materials for FDM; contrast the treating effect of nano CaCO_3_, CNF, and MCC on performance of BP/PLA composite filament using the impregnation modification technology. The mechanical properties and interfacial properties of the composite filaments were examined using the mechanical property tests, dynamic mechanical analysis, rheological tests, and environmental scanning electron microscope (ESEM) examination.

## 2. Materials and Methods

### 2.1. Materials

Nano calcium carbonate (CaCO_3_, 15–40 nm grain diameter, NCCa40) was supplied by Beijing Boyu Gaoke New Material Technology Co., Ltd., Beijing, China. Micro-crystalline cellulose (MCC, A17730), with a particle size of (1% + 60 mesh (250 μm) and <30% +200 mesh (75 μm)) and a bulk density of 0.25–0.35 g/cc, whose degree of polymerization is less than 350, supplied by Alfa Aesar (by Thermo Fisher Scientific, Shanghai, China). Cellulose nanofibers (CNF, 1.089 mmol/g carboxyl content), with a diameter of 10 nm and a length of 2 μm, was prepared through TEMPO-mediated oxidation from NBKP, which was bought from Tianjin Haojia Nano-crystalline Cellulose Co., Ltd., Tianjin, China. Bamboo powder (BP) was obtained from Zhejiang Tenglong Bamboo Group Co. Ltd., Quzhou, China, and the particle size distribution of BP was presented in Table 1. Polylactic acid (PLA, 4032D), with a density of 1.24 g/cm^3^ and melt flow index (MFI) of 7 (g/10 min–@210 °C/2.16 kg), supplied by NatureWorks (Blair, NE, USA). Analytically pure ethylenediamine tetraacetic acid disodium salt (EDTA-2Na) was purchased from Beijing Huabo Stand Biological Analysis Technology Co., Ltd., Beijing, China.

### 2.2. Preparation of Modified Bamboo Powder

Four specimen groups of BP were prepared for the experiment. The first specimen group was unmodified BP which was used as a control group. The other three specimen groups of BP were subjected to the impregnation modification process and were treated by nano CaCO_3_, MCC, or CNF, respectively. At 25 °C, 100 g of BP were dissolved in 2000 mL of deionized water for 30 min at 60 rpm, 1.7 g of EDTA-2Na, and 20 g of nano CaCO_3_, MCC, or CNF were then added to the mixture, which was then mixed for an additional 25 min. The suspensions were then rinsed on a 200-nylon mesh net with deionized water and then air dried. The modified bamboo powder (MBP), which includes nano CaCO_3_-BP, MCC-BP, and CNF-BP were obtained and preserved in a constant temperature and climate box at 23 °C and 50% relative humidity (RH).

### 2.3. Composite Filaments Processing: Preparation of PLA-Based Composite Filaments by the “One-Step” Method and “Two-Step” Method

In order to remove all absorbed moisture and prevent void formation, the BPs (particle size <74 μm), nano CaCO_3_-BPs, MCC-BPs, and CNF-BPs were dried at 103 °C in an oven until the moisture content (MC) was less than 2 wt%. The PLA was first dried in an oven at 80 °C for 4 h, then at 100 °C for 4 h, and lastly at 110 °C for 20 h before processing. The composite filaments were prepared by the following steps:

First, using a high-speed mixer, 20 wt% of BP or MBP (e.g., nano CaCO_3_-BP, MCC-BP, and CNF-BP) and 80 wt% of PLA were added and mixed fully.

**Figure 1:**
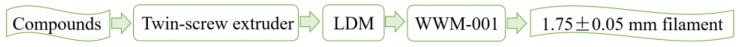
The composite filaments prepared by the “one-step” method.

**Figure 2:**
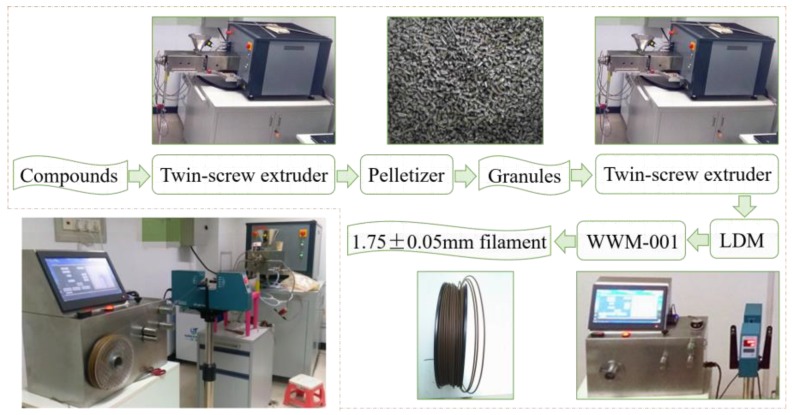
The composite filaments prepared by the “two-step” method.

Second, in this study, two methods were used to manufacture the composite filaments, that is, the “one-step” method and “two-step” method. (1) “One-step” method (Figure 1): the composite filament was manufactured by placing the mixture directly in an intermeshing counter-rotating conical twin-screw extruder (PolyLab QC, HAAKE, Karlsruhe, Germany); (2) “Two-step” method (Figure 2): the mixture was granulated using twin-screw extruder. The mixture underwent melt mixing in the twin-screw extruder and then was passed through the die of the extruder forming the composites. The composites were then pelletized in a pelletizer machine to produce the granules. The granules obtained from the pelletizer machine were then placed in the twin-screw extruder with a die diameter of 3 mm; where they were subjected to melt mixing and then water cooling to produce the composite filaments. In addition, the process was carried out at a temperature difference range of 175 °C, 175 °C, 170 °C between the feeding zone to die zone.

At last, the composite filaments with diameters of 1.75 ± 0.05 mm were obtained by using a laser diameter measuring (LDM) gauge (Mercury-Tech) and the wiredrawing-winding machine (WWM-001, independent research and development).

### 2.4. Rheological Studies

The melt viscosity of the composites was studied using a HAAKE Polylab torque rheometer (HAAKE, Karlsruhe, Germany) equipped with a Rheomix 600 QC counter-rotating roller rotors mixing chamber. The mixing chamber was loaded at 70% volume capacity and all measurements were performed over a constant revolution speed 40 rpm at a temperature of 170 °C.

### 2.5. Tensile Properties

Tensile tests were carried out according to ASTM D 638–2010 using an Instron universal testing machine (Instron, Norwood, MA, USA). Tests were performed with a load cell of 500 N and a cross-head speed of 2 mm/min on composite filament at room temperature (25 °C). The reported data were tensile strength (TS) and elongation at break (EAB). A minimum of five samples were tested for each formulation to get an average and standard deviation.

### 2.6. Dynamic Mechanical Analysis (DMA)

The storage modulus (*E*’), loss modulus (*E*’’), and loss factor (tan *δ*) of composite filaments were carried out according to ASTM D 7028-07^ε1^ using a dynamic mechanical analyzer (DMA Q800, TA Instruments, New Castle, PA, USA). DMA samples were vibrated with a tensile fixture at a frequency of 1 Hz. The samples were subjected to an amplitude of 15 μm in a temperature range of −20 °C to 120 °C at a heating rate of 2 °C/min.

### 2.7. Morphology Observation

The instrument used to analyze the surface morphology of the composite filaments and the interfacial quality between phases was a field emission environmental scanning electron microscope (ESEM; XL30 ESEM-FEG; FEI Co., Philips, The Netherlands). The samples were sputter coated with a thin layer of gold in a vacuum chamber for conductivity before the examination and were analyzed in a vacuum chamber that was less than 5 × 10^−5^ Pa at a voltage of 7 kV.

## 3. Results and Discussions

### 3.1. Rheological Properties

**Figure 3:**
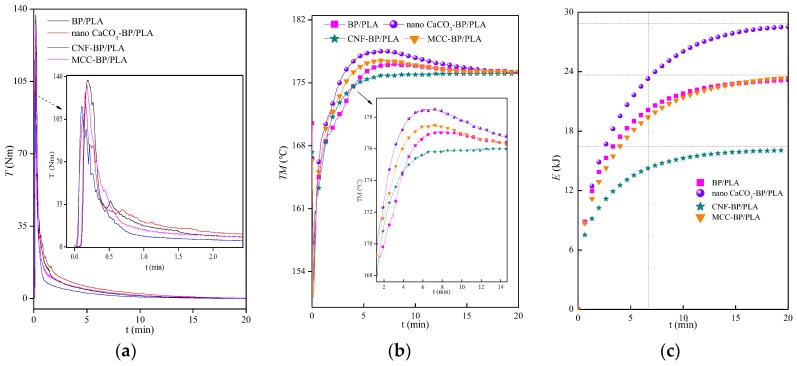
Torque *T* (**a**), melt temperature *TM* (**b**), and total energy *E* (**c**) of nano-particles reinforced bamboo powder/reinforced polylactic acid (BP/PLA) composite filaments vary with the time.

Interfacial bonding between the reinforcement and matrix plays a vital role in determining the mechanical properties of the composites and the interfacial interactions of the composites can be reflected by rheology tests. Rheology investigates the flow of matter via recording the change in the parameters such as torque (*T*), melt temperature (*TM*), and total energy (*E*) at a constant rotational speed. In general, the polymer melts exhibit non-Newtonian viscosity. Thus, aiming to investigate the effect of CNF, MCC, and nano CaCO_3_ on the rheological properties of composites in this study. Figure 3 showed a typical *T*-t, *TM*-t, and *E*-t plots of the blends test (e.g., BP and PLA, CNF-BP and PLA, MCC-BP and PLA, nano CaCO_3_-BP and PLA). There was a high initial *T* value during the fusion of all the polymers followed by a sharp decrease during the 20 min test. The *TM* of all the polymers rose sharply and then was followed by a general decrease, which is because the energy input reached a maximum value and the degradation of polymer reduced the viscosity during polymer melting and mixing process. As can be seen from Figure 3 that the balance torque *T*_bal_ and the maximum melt temperature *TM*_max_ (the balance melt temperature *TM*_bal_) were 0.2 Nm and 177.10 °C (176.2 °C), 0.2 Nm and 178.5 °C (176.2 °C), 0.1 Nm and 176.2 °C (176.2 °C), 0.3 Nm and 177.5 °C (176.3 °C) for BP/PLA, nano CaCO_3_-BP/PLA, CNF-BP/PLA, and MCC-BP/PLA composite, respectively. It can be noted that the *T*_bal_, *TM*_max_, and *TM*_bal_ values of all the composites did not see a significant change. In addition, the *T*_max_ increased from 115.6 Nm (CNF-BP/PLA) by 18.69% (to 137.2 Nm), 9.17% (to 126.2 Nm), 14.88% (to 132.8 Nm) while the maximum total energy *E*_max_ increased from 16.06 kJ (CNF-BP/PLA) by 44.33% (to 23.18 kJ), 77.77% (to 28.55 kJ), and 45.70% (to 23.4 kJ) for BP/PLA, nano CaCO_3_-BP/PLA, and MCC-BP/PLA composite, respectively. BP treated by CNF impregnation modification was the easiest to mix with PLA, which is due to a lower *T*_max_ and *E*_max_. It can be inferred that the nano CaCO_3_, CNF, and MCC each has a different impact on the rheological properties, suggesting that the incorporation of nano-particles into BP reinforced PLA composite filaments can result in disparate dispersion in the reinforcing phase due to the nanoscale dispersion and unique characteristics of the nano-particles. The good dispersion nano-particles can decrease the void numbers and provide strong interfacial hydrogen bond, thus developing better interfacial adhesion and improving the tensile properties of the composite filaments. It also can be inferred from these results that adopting nano CaCO_3_ to treat BP was better than using MCC and CNF to treat BP on the basis of the impregnation modification method.

### 3.2. Tensile Properties of Composite Filaments

**Figure 4:**
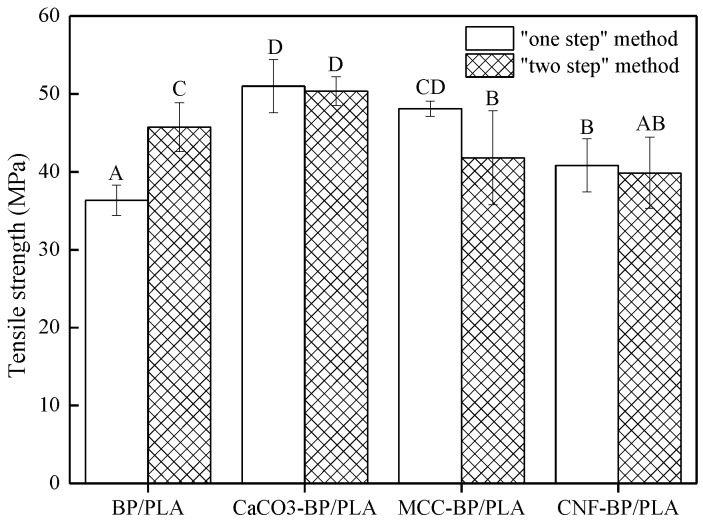
The tensile strength (TS) of reinforced polylactic acid (PLA)-based composite filaments. Note: There are significant differences at the 0.05 level of Duncan test and groups with the same letters do not differ statistically (*p* < 0.05).

The tensile strength (TS) results of BP/PLA, nano CaCO_3_-BP/PLA, MCC-BP/PLA, and CNF-BP/PLA composite filaments are shown in Figure 4. When the composite filaments were prepared by “one-step” method, the TS of nano CaCO_3_-BP/PLA, MCC-BP/PLA, and CNF-BP/PLA composite filaments increased by 40.33%, 32.35%, and 12.32%, respectively, compared with BP/PLA composite filaments. The TS enhancement observed in these composite filaments can be explained by the formation of a percolating network in the polymer matrix, in which stress transfer is facilitated by hydrogen bonding. The hydrogen bonding was best exemplified in the paper where these secondary interactions provide the basis of its mechanical strength. Furthermore, well-dispersed nanometer-sized elements in the polymer matrix may also serve as nucleating agents in the foaming process [31]. Moreover, crystalline cellulose is much stronger and stiffer than the amorphous cellulose and cellulose itself, which means that MCC can be a better reinforcing agent than cellulose [32]. Khalia et al. [33] also reported that MCC with high crystallinity can deliver a strong reinforcing ability because of the high modulus, which is capable of improving the mechanical properties of biocomposites. However, when the composite filaments were manufactured by the “two-step” method, the TS of nano CaCO_3_-BP/PLA increased by 10.10%, while MCC-BP/PLA and CNF-BP/PLA composite filaments both decreased by 8.61% and 12.85%, respectively. This is due to the fact that MCC and CNF existed in a net structure, which will reunite easily and sturdily because of the strong hydrogen bonding, which can affect the reinforcing effect. From Figure 4, it can be observed that except BP/PLA composite filaments, the TS result values of nano CaCO_3_-BP/PLA, MCC-BP/PLA, and CNF-BP/PLA composite filaments manufactured by the “one-step” method were slightly higher than those produced by “two-step” method. These higher TS values are due to the preparation process of the composite filaments, which causes PLA to degrade. The molecular chain of MCC and CNF contains a large number of hydroxyl groups, which can make PLA degrade enormously. It should be noted that CNF has more surface hydroxyl groups than MCC [34], which resulted in CNF-BP/PLA composite filaments having lower TS values than MCC-BP/PLA composite filaments. It also can be inferred that the effect of nano CaCO_3_ impregnation modification on TS of composite filaments was better than MCC and CNF impregnation modification.

**Figure 5:**
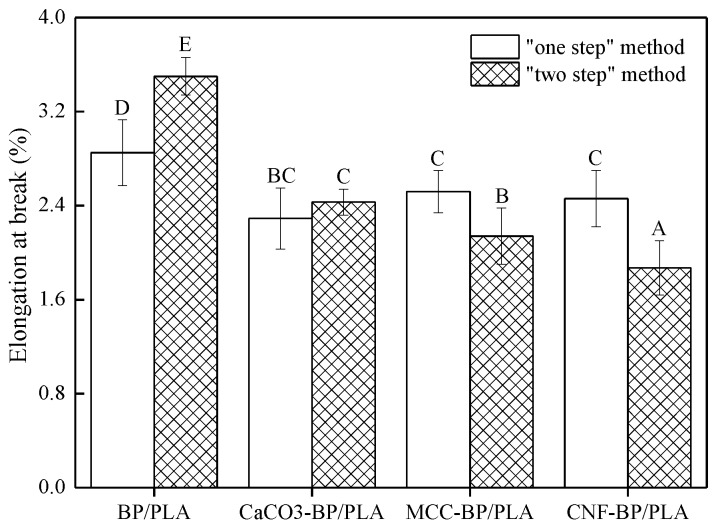
The elongation at break (EAB) of PLA-based composite filaments. Note: There are significant differences at the 0.05 level of Duncan test and groups with the same letters do not differ statistically (*p* < 0.05).

Figure 5 illuminates the elongation at break (EAB) of BP/PLA, nano CaCO_3_-BP/PLA, MCC-BP/PLA, and CNF-BP/PLA composite filaments. Having used any of the fore-mentioned preparation methods, the EAB of BP/PLA composite filaments was higher than that of BP/PLA treated by nano CaCO_3_ or MCC or CNF. This is due to the formation of nano CaCO_3_, MCC, and CNF, which act as stress concentrators that lead to an increase of brittleness, which in turn reduces the EAB. Moreover, nano CaCO_3_ or MCC or CNF probabilistically come into contact with BP directly, leading to stress cracks that propagated much more easily through the material [35]. Compared to nano CaCO_3_-BP/PLA composite filaments manufactured by the “one-step” method, the EAB of MCC-BP/PLA and CNF-BP/PLA composite filaments were increased by 10.04% and 6.99%, respectively, this is because the melt plasticization process of MCC-BP/PLA and CNF-BP/PLA composite filaments during the “one-step” method process was better than that of nano CaCO_3_-BP/PLA composite filaments. It also can be seen that the EAB of MCC-BP/PLA and CNF-BP/PLA composite filaments manufactured by the “two-step” method, decreased by 11.93% and 23.05%, respectively, in comparison to nano CaCO_3_-BP/PLA composite filaments, indicating that MCC and CNF made the PLA in the composite filaments manufactured by the “two-step” degrade much more than in CaCO_3_.

### 3.3. Dynamic Mechanical Properties

**Figure 6:**
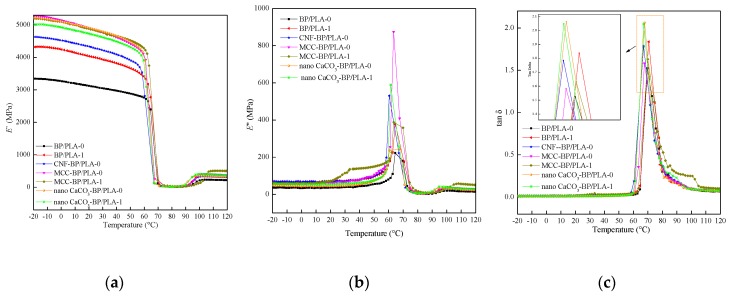
Storage modulus *E*’ (**a**), loss modulus *E*’’ (**b**) and loss factor tan *δ* (**c**) of composite filaments. Notes: The BP/PLA-0, nano CaCO_3_-BP/PLA-0, micro-crystalline cellulose (MCC)-BP/PLA-0, cellulose nanofibers (CNF)-BP/PLA-0 composite filaments were manufactured by the “one-step” method (Figure 1); and BP/PLA-1, nano CaCO_3_-BP/PLA-1, MCC-BP/PLA-1, and CNF-BP/PLA-1 composite filaments were manufactured by the “two-step” method (Figure 2).

The storage modulus *E*’, loss modulus *E*’’ and loss factor tan *δ* of the composite filaments as a function of temperature were determined by DMA and are shown in Figure 6. The DMA plots clearly revealed that the storage modulus *E*’ of the nano CaCO_3_-BP/PLA, MCC-BP/PLA, and CNF-BP/PLA composite filaments increased compared with the BP/PLA composite filaments at the temperature ranging from −20 °C to the glass transition temperature (*T*_g_). The reinforcement effect of nano CaCO_3_, MCC, and CNF gradually became less apparent when the temperature was above *T*_g._ This is because the rigid nanoparticles (e.g., nano CaCO_3_, CNF, and MCC) further limited the movement of the matrix molecular segments, thus causing the improvement in rigidity of the composite filaments. The data showed a remarkable modulus enhancement in the glassy state, where at −20 °C, *E*’ increased from 3.345 GPa (BP/PLA-0) by 56.17% (to 5.224 GPa), 58.03% (to 5.286 GPa), 38.48% (to 4.632 GPa) for nano CaCO_3_-BP/PLA-0, MCC-BP/PLA-0, and CNF-BP/PLA-0 composite filaments, respectively; while *E*’ increased from 4.322 GPa (BP/PLA-1) by 16.06% (to 5.016 GPa) and 20.34% (to 5.201 GPa) for nano CaCO_3_-BP/PLA-1 and MCC-BP/PLA-1 composite filaments, respectively. It also can be seen that the *E*’_−20°C_ of nano CaCO_3_-BP/PLA-0, and MCC-BP/PLA-0 composite filaments were a slight proportion higher than that of nano CaCO_3_-BP/PLA-1 and MCC-BP/PLA-1 composite filaments, respectively, which is consistent with the TS results. As can be seen from Figure 6b that the *E*’’ of the nano CaCO_3_-BP/PLA, MCC-BP/PLA, and CNF-BP/PLA composite filaments were higher than that of BP/PLA-0, which means that the large friction occurred due to mutual movement. It can be inferred that the energy of the thermally activated molecular movement was different for all the composite filaments. The tan *δ*_max_ value of BP/PLA-0 composite filaments were lower than nano CaCO_3_-BP/PLA, MCC-BP/PLA, and CNF-BP/PLA composite filaments (Figure 6c), which indicated that the CaCO_3_, MCC, and CNF had an effect on the movement of the PLA chain segments. Moreover, the crystallinity (*CrI*) of material can affect the dynamic mechanical properties, as it can be seen in the DMA curves [36], an increase in both storage and loss modulus in the temperature range of 90–120 °C was observed, this is due to the re-crystallization exotherm of PLA, leading to the crystals formed during the cold crystallization of PLA around 95 °C [37]. Additionally, it can be seen that the data of CNF-BP/PLA-1 (two-step method) composite filaments were not shown in Figure 6. This is because the EAB of CNF-BP/PLA-1 composite filaments was lower than the other materials, causing the higher brittleness, thus leading to the breakage which happened during the test. And according to the literature [38], the existence of CNF can improve the degradation rate from the beginning of degradation rather than in the process of degradation, which obviously improved the degradation of PLA.

### 3.4. Morphology Observation

**Figure 7:**
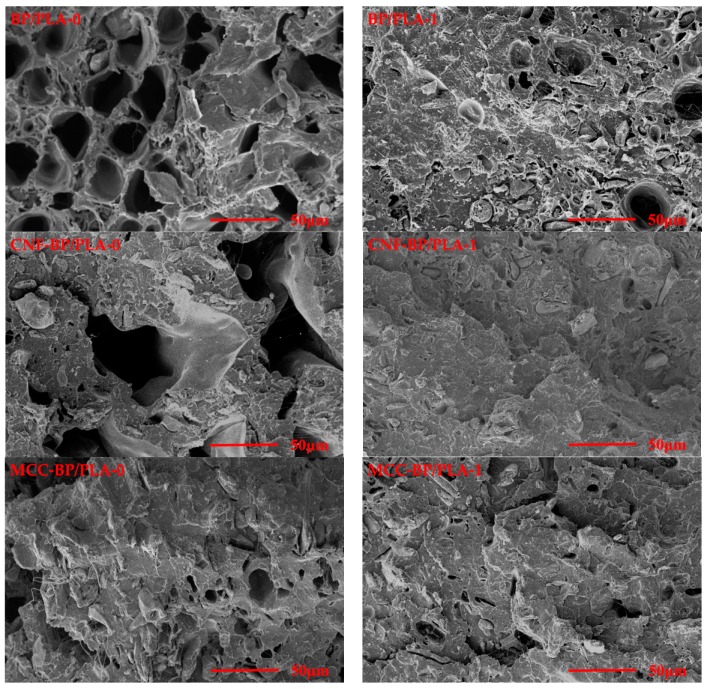

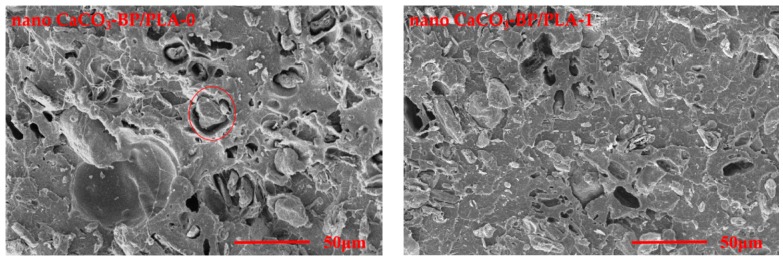
The tensile fracture surface of composite filaments. Notes: The BP/PLA-0, nano CaCO_3_-BP/PLA-0, MCC-BP/PLA-0, and CNF-BP/PLA-0 composite filaments were manufactured by the “one-step” method (Figure 1); and BP/PLA-1, nano CaCO_3_-BP/ PLA-1, MCC-BP/PLA-1, and CNF-BP/PLA-1 composite filaments were manufactured by the “two-step” method (Figure 2).

Typical environmental scanning electron micrographs (ESEM) of the tensile fractured surfaces were presented in Figure 7. These figures were used to investigate the interface quality between nano-particles (e.g., nano CaCO_3_, MCC, and CNF), BP, and PLA.

Figure 7 showed the corresponding ESEM images of BP/PLA, CNF-BP/PLA, MCC-BP/PLA, and nano CaCO_3_-BP/PLA composite filaments. Obviously, the level of interface adhesion between the reinforcement and matrix was reflected qualitatively by the ESEM micrographs. It can be seen that strong interface adhesion between modified BP (nano CaCO_3_-BP, MCC-BP, and CNF-BP) and matrix PLA was obtained since less space or voids between both phases can be observed. During failure at the interface, the BP may not be able to support a load and could be easy to pull out from matrix PLA. It also can be noticed that the reinforcing phases broke under tensile tests except for the reinforcement pullout, which indicated that the load was transferred from the PLA matrix to the reinforcements effectively. It can be inferred that the development of interface bonding was a sign of the tensile properties’ improvement in the composite filaments. The better interfacial interaction increased the tensile properties of the composite filaments manufactured by the “one-step” method, which was confirmed by the tensile testing results. In addition, according to the research [39,40], the tensile properties were related not only to the interfacial compatibility but also to the raw materials themselves. For nano-particles reinforced composites manufactured by the “two-step” method, the effect of raw materials on tensile strength was more than the interfacial compatibility between reinforcement and matrix. It also can be observed that the “two-step” method made the matrix PLA of nano-particles reinforced composite filaments decrease much more than the “one-step” method, thus, decreasing the molecular weight of PLA. However, this phenomenon did not happen for the BP/PLA composite filaments.

## 4. Conclusions

Based on our main objective to produce natural fiber reinforced composite materials with strong interfacial adhesion for 3D printing, especially for fused deposition modeling, this work introduced nano-particles impregnation modification. In summary, the BP/PLA composite filaments were treated with nano CaCO_3_, CNF and MCC using impregnation modification and were successfully produced using both a “one-step” and “two-step” method. The TS of the composite filaments manufactured by the “one-step” method were remarkably improved by adding nano CaCO_3_, CNF, and MCC. The TS of MCC-BP/PLA and CNF-BP/PLA composite filaments manufactured by the “two-step” method decreased due to the serious degradation of PLA. The TS of nano CaCO_3_-BP/PLA, MCC-BP/PLA, and CNF-BP/PLA composite filaments manufactured by the “one-step” method was slightly higher than those produced by the “two-step” method. The EAB of nano CaCO_3_-BP/PLA, MCC-BP/PLA, and CNF-BP/PLA was lower than that of the BP/PLA composite filaments, which is due to the increase in brittleness caused by impregnation modification. The *E*’ of the composite filaments increased through the addition of nano CaCO_3_, CNF, and MCC at the temperature ranging from −20 °C to the *T*_g_, while the reinforcement effect of nano CaCO_3_, MCC, and CNF gradually became less apparent when the temperature was above *T*_g_. The nano CaCO_3_-BP, MCC-BP, or CNF-BP and PLA were readily accessible by the simple mixing process. A detailed rheological investigation of such mixtures showed that the nano CaCO_3_, CNF, and MCC had a different impact on the processability and rheological properties of the composites. The effect of nano CaCO_3_ on the properties of composite filaments was better than MCC and CNF impregnation modification. 

## Figures and Tables

**Table 1:** The particle size distribution of bamboo powder (BP).

Particle size	<74 μm (>200 Mesh)	74~124 μm (120–200 Mesh)	124~178 μm (80~120 Mesh)	>178 μm (<80 Mesh)
Proportion	33.75%	16.53%	45.56%	4.15%
